# Effective treatment of experimental U-87MG human glioblastoma in nude mice with a targeted cytotoxic bombesin analogue, AN-215

**DOI:** 10.1038/sj.bjc.6600235

**Published:** 2002-04-22

**Authors:** Z Szereday, A V Schally, A Nagy, A Plonowski, A M Bajo, G Halmos, K Szepeshazi, K Groot

**Affiliations:** Endocrine, Polypeptide and Cancer Institute, Veterans Affairs Medical Center, 1601 Perdido Street, New Orleans, Louisiana, LA 70112-1262, USA; Section of Experimental Medicine, Department of Medicine, Tulane University School of Medicine, 1601 Perdido Street, New Orleans, Louisiana, LA 70112-1262, USA

**Keywords:** tumour targeting, doxorubicin, bombesin receptor, RT–PCR

## Abstract

Some brain tumours, such as glioblastomas express high levels of receptors for bombesin/gastrin releasing peptide. We investigated whether bombesin/gastrin releasing peptide receptors found in glioblastoma cell lines can be utilised for targeting of a cytotoxic bombesin analogue, AN-215 consisting of a potent derivative of doxorubicin, 2-pyrrolino-doxorubicin (AN-201) linked to a bombesin-like peptide carrier. This study reports the effect of AN-215 on the growth of U-87MG human glioblastomas xenografted into nude mice. High affinity binding of AN-215 to U-87MG tumours was characterised by an IC_50_ value of 4.0±0.1 nM, as determined by radioreceptor assays. mRNA analyses revealed the presence of mRNA for BN receptor subtypes 1 and 2. Treatment with AN-215 significantly (*P*<0.05) extended tumour doubling time from 4.54±0.2 days to 8.18±1.8 days and inhibited tumour growth as demonstrated by a 69.6% reduction in final tumour volume (*P*<0.001) and a 64.6% decrease in tumour weight as compared to controls. Cytotoxic radical AN-201 at the same dose was ineffective. The antitumour effect of AN-215 could be blocked by pretreatment with an excess of a bombesin antagonist, indicating that the action of this cytotoxic analogue is receptor-mediated. Our results suggest that patients with inoperable brain tumours such as malignant gliomas may benefit from targeted chemotherapy based on cytotoxic bombesin analogue AN-215.

*British Journal of Cancer* (2002) **86**, 1322–1327. DOI: 10.1038/sj/bjc/6600235 www.bjcancer.com

© 2002 Cancer Research UK

## 

About 17 000 Americans develop primary brain tumours and 13 000 die from them each year (Greenlee *et al*, 2000). The annual incidence rates of primary brain tumours in the United Kingdom, France, Norway and Finland and mortalities per 100 000 population are similar to those in the United States (Muir *et al*, 1994; Fleury *et al*, 1997; Prados and Levin, 2000). The incidence of brain cancer may be increasing in older people (Muir *et al*, 1994). Glioblastoma multiforme is the most common type of primary malignant CNS tumours in adults and is considered incurable (Hochberg and Pruitt, 1987).

Current treatment options for malignant gliomas, including surgery, radiation, and chemotherapy, are of limited effectiveness, and novel therapeutic modalities must be explored. Targeting of antineoplastic agents to cancer cells by linking them to a ligand with high affinity for tumour cells should improve tumour growth inhibition and decrease peripheral toxicity (Schally and Nagy, 1999). The presence of high affinity receptors for bombesin (BN)-like peptides on a wide variety of tumours prompted us to employ some of our powerful BN/gastrin releasing peptide (GRP) antagonists as carrier molecules for targeting cytotoxic agents to tumour cells (Nagy *et al*, 1997). One of these cytotoxic BN conjugates, AN-215 (Nagy *et al*, 1997) consists of a potent cytotoxic derivative of doxorubicin (DOX), 2-pyrrolino-DOX (AN-201) (Nagy *et al*, 1996), covalently linked through a glutaric acid spacer to the amino terminal of Gln-Trp-Ala-Val-Gly-His-Leu-ψ-Leu-NH_2,_ a BN-like peptide carrier (Nagy *et al*, 1997). AN-215 displays high affinity to BN receptors on Swiss 3T3 fibroblasts and retains the antiproliferative effect of its cytotoxic moiety (Nagy *et al*, 1997). This cytotoxic conjugate was shown to effectively inhibit BN receptor-positive neoplasms, including H-69 human small-cell lung carcinoma (Kiaris *et al*, 1999a) and PC-3 human androgen-independent prostate cancer (Plonowski *et al*, 2000).

In view of findings that 85% of human glioblastoma cell lines express functional receptors for BN/GRP (Moody *et al*, 1989; Staley *et al*, 1993; Pinski *et al*, 1994; Sharif *et al*, 1997), we evaluated in this study the efficacy of targeted chemotherapy based on cytotoxic analogue of BN, AN-215 in U-87MG human glioblastoma xenografted into nude mice.

## MATERIALS AND METHODS

### Peptide and cytotoxic agents

Cytotoxic conjugate AN-215 was made by coupling one molecule of 2-pyrrolino-DOX-14-*O*-hemiglutarate to the amino terminus of BN-like carrier analogue Gln-Trp-Ala-Val-Gly-His-Leu-ψ(CH_2_-NH)-Leu-NH_2_ as described (Nagy *et al*, 1997). GRP(14-27), cytotoxic radical AN-201 (Nagy *et al*, 1996) and BN/GRP antagonist RC-3095 [D-Tpi^6^,Leu^13^ψ(CH_2_NH)Leu^14^]BN (6-14) (Radulovic *et al*, 1991) were also synthesised in our laboratory. For intravenous (i.v.) injection, the compounds were dissolved in 20 μl of 0.01 N acetic acid and diluted with 5% (w v^−1^) aqueous D-mannitol (Sigma, St. Louis, MO, USA) solution.

### Cell lines, animals and tumours

Human glioblastoma cell line U-87MG was obtained from American Type Culture Collection (Manassas, VA, USA) and cultured as described (Pinski *et al*, 1994). Male athymic (Ncr nu/nu) nude mice, approximately 6-weeks-old on arrival, were obtained from the National Cancer Institute (Frederick Cancer Research and Development Center, Frederick, MD, USA), and housed in laminar air-flow cabinets under pathogen-free conditions with a 12 h light/12 h dark schedule, and fed autoclaved standard chow and water *ad libitum*. Xenografts were initiated by s.c. injection of 12×10^6^ U-87MG cells into the right flanks of five male nude mice. Tumours resulting after 2 weeks were aseptically dissected, mechanically minced, and 3-mm^3^ pieces of tumour tissue were transplanted s.c. with a trocar needle. The take rate was 100%. All experiments were performed in accordance with institutional ethical guidelines of animal care and were essentially in agreement with UKCCCR guidelines (1998) for the welfare of animals in experimental neoplasia.

### Experimental protocols

Experiment 1 was started when tumours had grown to approximately 40 mm^3^ in volume. Animals were randomly divided into three treatment groups: group 1(10 mice), control, which received vehicle solution; group 2 (10 mice), injected with cytotoxic radical AN-201; group 3 (11 mice), given cytotoxic BN analogue AN-215. Cytotoxic compounds were injected through the jugular vein at a dose of 150 nmol kg^−1^ of body weight (BW) on days 1, 8, 15 and 22. Tumour volume (length×width×height×0.5236) and BW were measured twice a week. The experiment was terminated on day 29. Blood samples were collected from the inferior vena cava under Metofane (Malinkrodt Vet. Mundelein, IL, USA) anaesthesia and then Metofane was used in overdose to sacrifice the mice. Tumours were excised and weighed. Tumour specimens were snap-frozen and stored at −70°C until extraction of RNA for reverse transcription-polymerase chain reaction (RT–PCR). Tumour volume doubling time was calculated between day 1 and 29 using the formula:


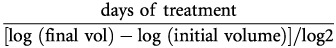


as described by Smolev *et al* (1977).

Experiment 2 was started when U-87MG tumours had grown to 70–84 mm^3^ in volume. The animals were divided into the following treatment groups: group 1 (10 mice), control, vehicle solution; group 2 (10 mice), analogue AN-215; group 3 (five mice), unconjugated mixture of the cytotoxic radical AN-201 and BN antagonist RC-3095. All compounds were injected i.v. at 150 nmol kg^−1^ of BW on days 1, 10, and 17. In addition, one group of tumour-bearing mice received an i.v. injection of 200 μg of BN antagonist RC-3095 15 min before each administration of AN-215 at a dose of 150 nmol kg^−1^ of BW on days 1, 10 and 17 as in the case of group 2. The experiment was terminated on day 20 as described above.

### Evaluation of toxicity

General toxicity was evaluated on the basis of mortality rate and changes in BW. Toxicity to BN receptor-positive organs was assessed by measuring gastrin release in response to GRP stimulation (Plonowski *et al*, 2000). At the end of experiment 1, blood samples were collected 5 min after i.v. injection of 2 μg of GRP(14-27) dissolved in 100 μl of 5% mannitol. Serum gastrin concentrations were measured by double-antibody radioimmunoassay with a kit provided by ICN Pharmaceuticals Diagnostic Division (Orangeburg, NY, USA). The interassay variation was 10.6%, and the intraassay variation was 6%.

### Histological methods

Samples of tumour tissues were fixed in 10% buffered formalin. The specimens were embedded in Paraplast (Oxford Labware, St. Louis, MO, USA). Six μm thick sections were cut and stained with haematoxylin-eosin. Mitotic and apoptotic cells were counted in nine standard high power microscopic fields containing an average of 330 cells, and their numbers per 1000 cells were accepted as the mitotic and apoptotic indices, respectively. To eliminate errors caused by variances in thickness of slides and cellularity of areas investigated, the ratio of apoptotic to mitotic indices was calculated in each tumour. For the determination of the extent of necrosis in tumours, the crossing points of a microscope ocular net that coincided with necrosis in the slide made at the largest cross-section of each tumour were counted. The ratio of these points to the number of all points above the tumour represented the percentage area of necrosis. For demonstration of the nucleolar organiser region (NOR) in tumour cell nuclei, the AgNOR method was used (Szepeshazi *et al*, 1991). NORs are parts of DNA closely associated with nucleoli and encode for ribosomal RNA. They are also associated with argyrophylic proteins and thus can be visualised by silver staining. The amount of AgNORs is a good indicator of cell proliferation (van Diest *et al*, 1998). The silver-stained black dots in 50 cells of each tumour were counted and the AgNOR number per cell was calculated. The data were evaluated by one way analysis of variance (ANOVA) and the treated groups compared to the control by Dunnett's test.

### RNA extraction and RT–PCR

Total RNA was extracted from frozen tissue samples by using RNAzolB (Tel-Test, Friendswood, TX, USA) according to the manufacturer's instructions. Concentrations of total RNA were calculated by measuring the OD_260_ value. Total RNA (4 μg) was subjected to electrophoresis for 2 h at 70 V in 10 μl of sample buffer containing 1× MOPS buffer (pH 6.5), 6.4% formaldehyde, 48% deionized formamide, 0.25% w v^−1^ bromophenol blue, 0.05% glycerol and 0.25 mg ml^−1^ ethidium bromide. The RNA gel contained 1.4% agarose, 1×MOPS (pH 6.5) and 1.73% formaldehyde.

The RT–PCR was performed using Gene Amp RNA Core Kit (Perkin-Elmer, Foster City, CA, USA). To avoid genomic DNA contamination, all samples were subjected to DNase digestion before RT–PCR. RNA (2 μg) was treated with 0.3 unit of RQ1 RNase-free DNase (Promega, Madison, WI, USA) at 37°C for 30 min in 19 μl of mixture containing 5 mM MgCl_2_, 1× PCR buffer, 1 mM of each dNTP, 1 unit RNase inhibitor and 2.5 μM random hexamers. The reaction was terminated by enzyme denaturation at 99°C for 5 min. After cooling, 2.5 units of murine leukaemia virus (MuLV) reverse transcriptase was added and RT was carried out at 42°C for 30 min.

The PCR amplification of the cDNAs for human glyceraldehyde-3-phosphate dehydrogenase (hGAPDH), GRP receptor (hGRPR, BRS-1) and neuromedin-B receptor (hNMBR, BRS-2) was performed as follows. Two μl of the cDNA were amplified in a 25-μl solution containing: 2 mM MgCl_2_, 1× PCR buffer, 200 μM of each dNTP, 2.5 units of Ampli Taq DNA polymerase and 0.2 μM of each primer. The primers used were 5′- TCC TCT GAC TTC AAC AGC GAC ACC-3′ and 5′-TCT CTC TTC CTC TTG TGC TCT TGG-3′ for hGAPDH, 5′-ATT TGG CAG GAT TGG CTG C-3′ and 5′-TGA GGC AGA TCT TCA TCA G-3′ for hGRPR, 5′- CGG ACT CTG CTG GAA AGG A-3′ and 5′-GAC GTC TGC ATG TCC ATG G-3′ for hNMBR (Kiaris *et al*, 1999b). Samples were denatured at 94°C for 5 min and then subjected to 30 cycles for hGAPDH or 40 cycles for hNMBR of 94°C for 30 s, 60°C for 30 s and 72°C for 40 s or 35 cycles for hGRPR of 94°C for 1 min, 58°C for 1 min and 72°C for 1 min, followed by a final extension at 72°C for 7 min using a Perkin-Elmer DNA thermal cycler model 2400. The number of cycles was determined in preliminary experiments to be within the exponential range of PCR amplification. Negative controls using distilled water instead of cDNA in the PCR mixture were run in parallel to exclude genomic DNA contamination. Aliquots of each PCR product were subjected to electrophoresis on a 2% agarose gel, stained with ethidium bromide and visualised under ultraviolet light. For the quantitation of PCR-amplified products, a scanning densitometer (model GS-700, Bio-Rad) coupled with the Bio-Rad personal computer analysis software was used. All experiments were repeated at least twice and similar results were obtained. The relative mRNA levels of each gene were normalised *vs* the corresponding levels of hGAPDH.

### Receptor binding assays

Binding characteristics of BN receptors on membrane preparations from U-87MG tumours were determined by ligand competition assays using ^125^I-labelled [Tyr^4^]BN, as reported earlier (Halmos and Schally, 1997). Receptor binding affinity of cytotoxic BN analogue AN-215 to tumour membranes was measured in displacement experiments based on competitive inhibition of ^125^I-[Tyr^4^]BN binding, using various concentrations of AN-215 (10^−6^–10^−12^ M). IC_50_ value was calculated with a computerized curve fitting programme and is defined as the concentration of AN-215 causing a 50% inhibition of ^125^I-[Tyr^4^]BN binding.

### Statistical analysis

Data are expressed as mean±s.e. Differences between mean values were evaluated by two-tailed Student's *t*-test, *P*<0.05 being considered significant.

## RESULTS

### Inhibition of tumour growth by AN-215

**Figure 1:**
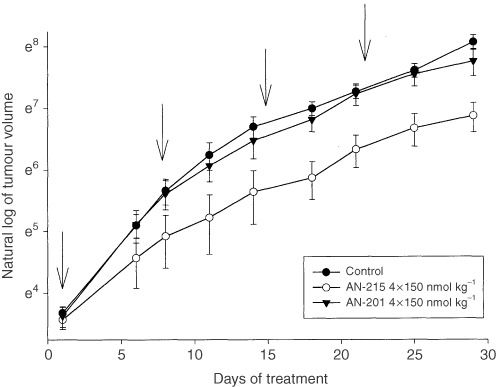
The effects of cytotoxic BN analogue AN-215 and cytotoxic radical AN-201 on the growth of s.c. xenografts of U-87MG human glioblastoma in nude mice (experiment 1). The treatment consisting of four i.v. injections of the respective compounds at 150 nmol kg^−1^ of BW, was started when tumour volume reached approximately 40 mm^3^ (arrows indicate the days of injections; vertical bars show s.e.).

Experiment 1 was designed to compare the antitumour effects and toxicity of cytotoxic BN analogue AN-215 and its cytotoxic radical AN-201. A treatment regimen consisting of four i.v. injections of AN-215 at 150 nmol kg^−1^ of BW produced a strong tumour growth inhibition (Figure 1

). The inhibitory effect of AN-215 was evident within 6 days and became significant from day 11. Four weeks after the initiation of the treatment, the tumour doubling time in animals treated with AN-215 was significantly prolonged from 4.5±0.2 to 8.2±1.8 days (*P*<0.05) (Table 1

**Table 1:**
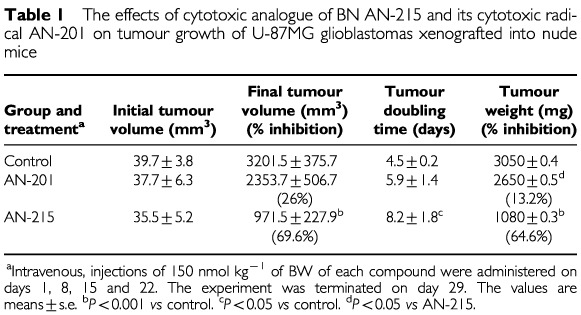
The effects of cytotoxic analogue of BN AN-215 and its cytotoxic radical AN-201 on tumour growth of U-87MG glioblastomas xenografted into nude mice

). In contrast, tumour doubling time in mice given an equimolar dose of AN-201 was 5.9±1.4 days which did not differ significantly from the controls. Tumour volume and weight are shown in Table 1.

**Figure 2:**
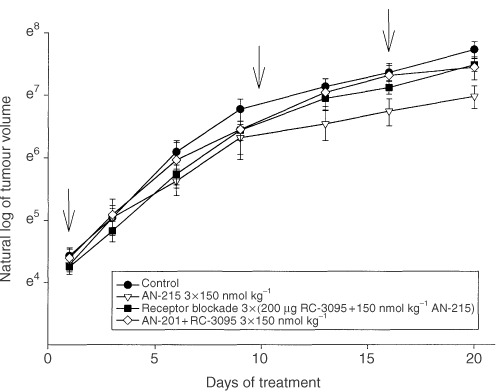
Evaluation of the antitumour effect of AN-215 in animals pretreated or not with 200 μg of BN antagonist RC-3095 15 min before administration of AN-215 at a dose of 150 nmol kg^−1^ to block the receptor-mediated uptake of the conjugate. Effects of an unconjugated mixture of cytotoxic radical AN-201 and RC-3095 are also shown. (Arrows indicate the days of injections; vertical bars show s.e.).

In experiment 2, the treatment was initiated when xenografts of U-87MG glioblastomas had grown to a volume, approximately twice as large as that in experiment 1. After 20 days the tumour doubling time in mice treated with three i.v. injections of AN-215 was extended significantly (*P*<0.05) to 6.6±1.0 days compared to the control group, which came to 4.5±0.2 days. The changes in tumour volume are presented in Figure 2

.

### Effect of blockade of BN receptors

As part of experiment 2, mice bearing U-87MG tumours were pretreated i.v. with a high dose of BN antagonist RC-3095 (200 μg/mouse) to block BN receptors prior to each administration of AN-215. As shown in Figure 2, the pretreatment with RC-3095 attenuated the antitumour effect of the cytotoxic BN analogue AN-215, the growth inhibition being reduced to only 22% and the tumour doubling time was 4.7±02 days, close to control data.

### Toxicity

In experiment 1, two of 10 animals (20%) died in the group that received AN-201, but only one of 11 animals (9%) died after treatment with AN-215. To estimate the side effects caused by targeting the cytotoxic agent to normal organs that express GRP/BN receptors, we evaluated the effect of AN-201 and AN-215 on the serum concentration of gastrin following i.v. stimulation with GRP(14-27) at the end of the experiment. Neither AN-215 nor AN-201 affected GRP-stimulated gastrin release (Table 2

**Table 2:**
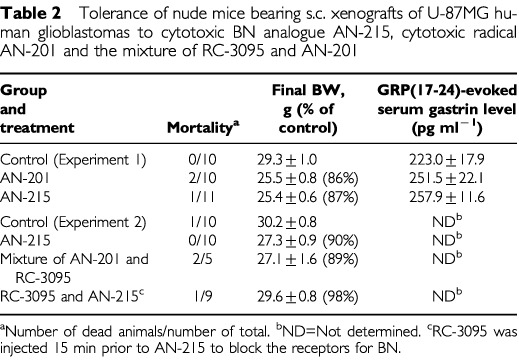
Tolerance of nude mice bearing s.c. xenografts of U-87MG human glioblastomas to cytotoxic BN analogue AN-215, cytotoxic radical AN-201 and the mixture of RC-3095 and AN-201

).

In experiment 2, two of five animals died (40%) in the group that received an unconjugated mixture of AN-201 and RC-3095 and one of nine animals (11%) died in the group that received an i.v. injection of 200 μg of BN antagonist RC-3095 about 15 min before administrations of AN-215, but no toxicity-related deaths occurred during the experiment in the AN-215-treated group. One of 11 animals (9%) died in the control group on the day when the experiment was terminated.

### Histology

Histologically, U-87MG glioblastomas are highly cellular tumours consisting of relatively large undifferentiated cells showing a moderate polymorphism. The cells have narrow cytoplasms and round or oval nuclei with loose chromatin structure and prominent nucleoli. At some areas, there are more elongated cells arranged in large bundles. The quantitative histological data are shown in Table 3

**Table 3:**
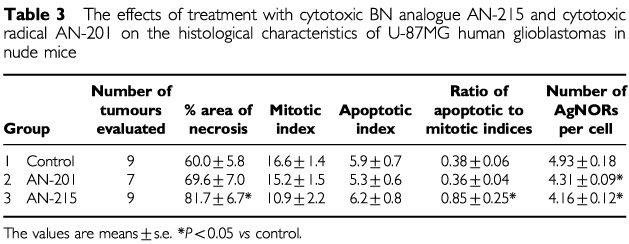
The effects of treatment with cytotoxic BN analogue AN-215 and cytotoxic radical AN-201 on the histological characteristics of U-87MG human glioblastomas in nude mice

. The extent of necrosis in the tumours treated with AN-215 was significantly larger than in the controls, while AN-201 caused only a slight and not significant change in necrosis. The mean mitotic and apoptotic indices were not appreciably changed by the treatments. However, a slight decrease in mitosis and an increase in apoptosis in the tumours treated with AN-215 resulted in a significant elevation of the ratio of apoptotic to mitotic indices.

### Expression of mRNAs for GRP/BN receptors

**Figure 3:**
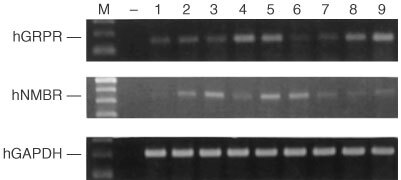
Agarose gel electrophoresis of reverse-transcribed and PCR-amplified mRNAs for hGRPR/BRS-1 and hNMBR/BRS-2 in U-87MG tumours in nude mice. The bands for the corresponding hGAPDH are also shown. M, DNA size marker; −, negative control; 1–3, untreated tumours; 4–6, AN-201-treated tumours; 7–9, AN-215-treated tumours. All PCR- amplification reactions yielded products of the expected size, which were 158 bp for hGRPR/BRS-1, 484 bp for hNMBR/BRS-2 and 207 bp for hGAPDH.

The total RNA isolated from control and treated samples was run on formaldehyde agarose gel. As expected, there were only two intense bands, corresponding to the 28S and 18S rRNAs indicating that the total RNA was intact (data not shown). The levels of mRNA for BN/GRP receptor subtypes hGRPR/BSR1 and hNMBR/BRS-2 were analysed in U-87MG tumours of all groups in experiment 1 (Figure 3

). Densitometric analyses of RT–PCR products revealed that hGRPR/BRS-1 mRNA expression was 15.1±1.5 arbitrary units (a.u.) in the control group and 21.6±3.4 a.u. and 25.4±5.1 a.u. in the AN-201- and AN-215-treated groups, respectively; the increase in both treated groups being not significant. The hNMBR/BRS-2 mRNA expression was 7.6±3.5 a.u. in the control group and 10.9±3.6 a.u. and 3.3±1.0 a.u. in the AN-201- and AN-215-treated groups, respectively. The decrease in the AN-215 group was again not statistically significant. No corresponding PCR products were detected from the negative control, indicating that the PCR products generated from cDNA, and not from genomic DNA contamination. PCR products with the expected size of 207 bp for hGAPDH gene were present in all samples (Figure 3) confirming that no RNA degradation occurred during the preparations.

### Radioreceptor assays

In the control group, radiolabelled [Tyr^4^]BN was bound to a single class of specific binding sites with high affinity (*K*_d_=5.36± 0.69 nM), and low capacity (B_max_=362.7±11.3 fmol mg^−1^ membrane protein). Specific high affinity binding sites for BN/GRP were also found on the AN-215-treated tumours (*K*_d_= 5.98±0.91 nM, B_max_=362.6±8.6 fmol mg^−1^ membrane protein). Thus, the treatment with AN-215 did not affect the binding characteristics of receptors for BN/GRP in U-87MG human glioblastoma.

The concentration of unlabelled AN-215 required to inhibit 50% of the specific ^125^I-[Tyr^4^]BN binding (IC_50_) was 4.0±0.1 nM. This IC_50_ value of AN-215 represents a high binding affinity to BN/GRP receptor protein expressed on U-87MG tumours.

## DISCUSSION

Chemotherapy is still one of the major modalities for the treatment of inoperable malignancies or for adjuvant therapy after surgery. However, the use of antineoplastic agents is hampered by their non-specific toxicity to normal, healthy tissues and intrinsic or acquired chemoresistance of cancerous cells. The targeting of chemotherapeutic agents specifically to tumour sites is a modern approach that may help overcome some of these drawbacks (Schally and Nagy, 1999).

Recently, we developed a series of targeted cytotoxic conjugates based on analogues of peptide hormones such as luteinizing hormone-releasing hormone(LH-RH), somatostatin and BN/GRP, for which high affinity receptors are expressed on a variety of tumours (Schally and Nagy, 1999; Nagy and Schally, 2001). DOX or its intensely potent derivative, 2-pyrrolino-DOX (AN-201), which is 1000 times more potent than DOX *in vitro*, were linked through their 14-*O*-glutaryl esters to a free amino group in the peptide carriers to form conjugates which retained cytotoxic activity and high binding affinity to respective receptors. LH-RH analogue AN-207 containing cytotoxic radical AN-201 also strongly inhibits DOX-resistant MX-1 human breast cancers *in vivo* (Kahan *et al*, 1999), indicating that 2-pyrrolino-DOX is non-cross-resistant with DOX, in analogy with similar highly potent derivatives of DOX such as Nemorubicin (Nagy and Schally, 2001). In addition, we demonstrated that the presence of receptors for somatostatin on U-87MG human glioblastomas can be utilised for an improvement in therapeutic efficacy when the targeted cytotoxic somatostatin analogue AN-238 which contains AN-201 is used, but not its counterpart containing DOX (Kiaris *et al*, 2000).

It has been shown that a high percentage of brain tumour cell lines, including U-87MG, express receptors for BN/GRP (Sharif *et al*, 1997). In our study we evaluated the effects of targeted therapy of U-87MG human glioblastoma xenografted into nude mice utilising one of our cytotoxic BN-like conjugates, AN-215 containing AN-201. The results of experiment 1 indicate, that similarly to somatostatin receptors, BN/GRP receptors on brain tumours can also be employed for the targeting of conjugates containing the highly potent derivative of DOX, AN-201 in order to improve the efficacy and lower its toxicity. This finding is in agreement with our previous results on PC-3 human androgen independent prostate cancers and H-69 small cell lung cancers, which indicated that improved efficacy can be achieved by targeting AN-201 to receptors for somatostatin or BN/GRP. To corroborate the concept that AN-215 acts through the binding to receptors for BN on U-87MG tumours, we designed a second experiment, in which an excess of the BN antagonist RC-3095 was injected i.v. 15 min before the administration of AN-215 in order to block the receptors for BN. As anticipated, the blockade of BN receptors significantly lowered the antitumour activity of AN-215 resulting only in a non-significant growth inhibition of U-87MG glioblastomas, similar to that produced by AN-201. This insignificant effect is probably due to the fact that the blockade of the BN receptors caused a longer exposure of AN-215 to non-specific carboxylesterase enzymes (EC3.1.1.) in the circulation, which can release the cytotoxic radical before the targeting is completed (Nagy *et al*, 2000). Preclinical and clinical results with new radioligands, developed for BN-receptor scintigraphy, also indicate that BN analogues can accumulate in BN receptor-positive tumours, further supporting the theory that BN receptors can be used for targeted chemotherapy (Breeman *et al*, 1999; Van de Wiele *et al*, 2000).

Binding assays using ^125^I-labelled[Tyr^4^]BN confirmed the presence of BN/GRP receptors on U-87MG tumour membranes (Pinski *et al*, 1994) and demonstrated a high affinity binding of AN-215 to these receptors characterised by an IC_50_ value of 4.0±0.1 nM. RT–PCR analyses also confirmed previous findings that these tumours express mRNAs for BN receptor subtype 1 and 2 but not for the orphan receptor subtype 3 (Kiaris *et al*, 1999b)

Histological analysis showed that treatment with AN-215, but not with cytotoxic radical AN-201, increased significantly the area of necrosis and the ratio of apoptotic to mitotic indices in U-87MG tumours. This can be explained by a higher accumulation of the cytotoxic radical in tumour tissue after treatment with AN-215, as a result of targeting. The histological data also suggest that such accumulation of AN-201 can cause cell death through necrosis, which may be preferable in cancer therapy to that caused by apoptosis (Kiaris and Schally, 1999).

In our previous study (Plonowski *et al*, 2000) we reported that after the final injection of AN-215, AN-201 or the mixture of AN-201 and RC-3094 there was an approximate 50% reduction in WBC count in nude mice bearing PC-3 human prostate cancers. This decrease in WBC was accompanied by a transient decrease in BW. In the present study no measurements of WBC were carried out. Instead, the assessment of toxicity was based on changes in BW and mortality which indicated, in both experiments, that targeted cytotoxic BN analogue AN-215 is not toxic (Table 2). In experiment 1, two of 10 animals (20%) died after four consecutive injections of AN-201 at 150 nmol kg^−1^ of BW dose but, one of 11 mice (9%) also died receiving the same dose of AN-215. This toxicity of AN-215 can be explained by the fact that the activity of esterase enzymes in the serum of nude mice is very high, about six times higher than in humans, causing a partial release of AN-201 before the targeting is completed (Nagy *et al*, 2000). This phenomenon can also account for the death of one of nine animals in experiment 2, in which a high dose of BN antagonist RC-3095 was injected into nude mice to block BN receptors, before each of the three injections of AN-215 at 150 nmol kg^−1^ of BW. The blockade of BN receptors would inhibit receptor-mediated accumulation of AN-215 in BN receptor-positive tissues, and the extended time of AN-215 in the circulation can cause the release of a high percentage of AN-201 from the conjugate. In this experiment, no deaths occurred in the group treated with the same dose of AN-215 without pretreatment with RC-3095. In further support of this view, a significantly higher tolerance to AN-215 by nude mice was demonstrated when carboxylesterase enzymes were inhibited (Nagy *et al*, 2000; Plonowski *et al*, 2000). Because receptors for BN are also expressed in various normal tissues such as the CNS, lungs, pancreas, breast, prostate or the gastrointestinal tract, it is assumed that there is a competition for targeted cytotoxic conjugate AN-215 between normal and cancerous tissues. Our previous results indicate that despite this competition, AN-215 is highly efficacious even in nude mice with suppressed esterase activity (Plonowski *et al*, 2000). In this study and also in our previous work (Plonowski *et al*, 2000), we found that AN-215 caused no specific damage to the gastrointestinal cells as determined by GRP-stimulated gastric secretion.

In conclusion, the present study demonstrates that cytotoxic BN analogue AN-215 is not toxic and significantly more effective in inhibiting the growth of U-87MG human glioblastomas than its non-targeted cytotoxic radical AN-201. Because receptors for BN/GRP are present on a high percentage of human glioblastoma cell lines, it is likely that further studies with AN-215 followed by therapeutic trials could result in the development of a new therapeutic approach for the management of patients suffering from advanced glioblastomas.
